# Age related cerebrospinal fluid flow dynamics in the subarachnoid space of the optic nerve in patients with normal tension glaucoma, measured by diffusion weighted MRI

**DOI:** 10.1038/s41433-024-03084-3

**Published:** 2024-04-25

**Authors:** Jatta Berberat, Achmed Pircher, Luca Remonda, Hanspeter E. Killer

**Affiliations:** 1 Institute of Neuroradiology, Kantonsspital Aarau, Aarau, Switzerland; 2 Department of Neuroscience/Ophthalmology, Uppsala University, Uppsala, Sweden; 3 Faculty of Medicine, University of Bern, Bern, Switzerland; 4 Department of Biomedicine, University of Basel, Basel, Switzerland; 5 Augenärzte Zentrum Aarau, Aarau, Switzerland

**Keywords:** Predictive markers, Predictive markers

## Abstract

**Background/objectives:**

We aimed to measure cerebrospinal fluid (CSF) flow rates in the subarachnoid space (SAS) of the optic nerve (ON) by applying non-invasive diffusion-weighted MRI in patients with normal tension glaucoma (NTG) compared to age-matched controls.

**Subjects/methods:**

In this prospective study, an analysis of diffusion-weighted images of 26 patients with NTG (49ONs) and age-matched volunteers (52ONs) was conducted. Subjects were classified into 4 groups: group I (50–59 y., *n* = 12 eyes), group II (60–69 y., *n* = 16 eyes), group III (70–79 y., *n* = 18 eyes) and group IV ( > 80 y., *n* = 6 eyes) for NTGs and healthy volunteers, respectively. The flow-range ratio (FRR) between the frontal lobe SAS and the SAS of the ON was calculated for each age category group and then compared between age-categories as well as between NTGs and controls.

**Results:**

The mean FRR for age groups were (I) 0.54 ± 0.06 and 0.62 ± 0.03 (*p* < 0.05), (II) 0.56 ± 0.08 and 0.63 ± 0.03 (*p* < 0.05), (III) 0.54 ± 0.06 and 0.62 ± 0.02 (*p* < 0.001) as well as (IV) 0.61 ± 0.03 and 0.61 ± 0.04, for NTGs and controls, respectively. Using pooled data, the difference between the FRR in NTGs and controls was statistically significant (*p* < 0.0001). There were no statistically significant differences within the age categories of the control group. When comparing the FRR of NTGs by age categories, no statistically significant difference was found between the subgroups.

**Conclusions:**

FRR was significantly reduced in NTGs compared to age-matched controls without any significant differences within the age groups themselves. Given the physiological importance of CSF for the integrity of neurons, axons and glial cells, reduced CSF flow dynamics might be part of the underlying neurodegenerative process of NTG.

## Introduction

Normal tension glaucoma (NTG) is thought to be a variant of primary open angle glaucoma (POAG). It is estimated to make up to 40% of POAG in the western hemisphere [1] and up to 90% in Asian countries [2]. NTG, like open angle glaucoma is more common in the elderly and rather unusual in patients younger than 50 years of age [3]. The average reported age in clinical studies is generally in the 60 s. Epidemiologically, age is therefore one of the main risk factors for NTG [4].

The role of intraocular pressure (IOP) in NTG is not well understood but lowering IOP has been shown to somehow slow the progression of visual field loss [5]. However, even after successful IOP lowering, retinal ganglion cell loss with consecutive visual field defects is progressive in many patients. To explain this phenomenon other pathophysiological mechanisms were introduced, among them vascular dysregulation, a higher than normal pressure gradient across the lamina cribrosa, glymphatic stasis within the optic nerve and compartmentation of cerebrospinal fluid (CSF) within the subarachnoid space (SAS) of the optic nerve [6, 7].

CSF functions as a transport system that delivers vital substances such as neurotransmitters, hormones, minerals and nutrients to the neuronal tissue such as neurons, axons and glial cells. On the other hand, it removes toxic metabolites from the parenchyma, among them abetalipoprotein and alfasynuclein from the parenchyma. Disturbed CSF dynamics lead to an undersupply of neurotrophic factors and to an accumulation of toxic biological substances [8, 9].

Recent studies [10] using cisternography and diffusion-weighted magnet resonance imaging (DWI MRI) [11] demonstrated decreased CSF flow along the ON in patients with NTG. This finding indicates that the CSF flow and thus CSF turnover along the optic nerve (ON) can become reduced in patients with NTG. Several pathophysiological processes, such as inflammation, elevated pressure and other age-related degenerative processes can cause a remodelling of the subarachnoid space (like arteriosclerotic changes in blood vessels) resulting in compartmentalisation of the SAS of the ON [12].

DWI provides a non-invasive measurement of the movement of molecules, mainly water in biological tissues. Using phase contrast images, the phase shift can be used to determine the flow velocities of coherently using particles [13, 14].

The aim of this study was to measure CSF flow rates in the SAS of the ON by applying non-invasive diffusion weighted MRI in patients with NTG compared to age-matched non glaucoma controls. Patients and controls were divided in age groups in order to investigate a possible influence of age on CSF flow rates.

## Materials and methods

This monocentric, prospective observational study was approved by the local ethical commission and followed the tenets of the Declaration of Helsinki. All studied individuals, age matched healthy controls, (*n* = 52 eyes, age range from 50 to 87 years) and NTG patients (*n* = 49 eyes, age range from 51 to 84 years), gave their written informed consent.

### Subjects

NTG was diagnosed on the basis of glaucomatous optic disc morphology and concomitant visual field defects with IOP always (without treatment) < 21 mmHg. Glaucomatous changes included optic disc cupping, with or without notches in the neuroretinal rim and localised or segmental loss of retinal nerve fibre layer. Visual field defects were shown by standard automated perimetry (SAP) (Programme G2, Octopus Haag-Streit, Switzerland).

Each patient underwent full ophthalmologic examination including slit lamp assisted biomicroscopy, applanation tonometry, gonioscopy, measurement of central corneal thickness and neuroretinal rim assessment with Heidelberg OCT (HEYEX, red sector on colour code compared to normative database, Heidelberg engineering, CA, USA). None of the patients had a refractive error > +3 or < −3 dioptres (D). 8/26 patients (6 bilateral, 2 unilateral) had IOP lowering drops to keep the IOP as low as possible. 12/26 patients underwent cataract surgery (10 bilateral, 2 unilateral) and none of them had glaucoma surgery. The mean glaucomatous visual field defect (MD) at time of MRI was 12 ± 6 dB and the mean IOP 12 ± 3 mmHg. None of the included NTG patients suffered from any other eye disease or underwent previous posterior segment surgery that may affect the visual field.

### Controls

All control subjects were volunteers and none of the controls had glaucomatous optic nerve morphology, nor were they on anti-glaucomatous treatment. For each healthy control, the anatomical MRI scan of the ON was normal.

All subjects (NTG and controls) were classified into 4 groups: group I (50–59 years old; n_NTG_ = 10 ONs, mean age: 53 ± 1 years, 6 males; n_controls_ = 12 ONs, 54 ± 3 years, 3 females), group II (60–69 years old; n_NTG_ = 15 ONs, mean age: 65 ± 3 years, 6 females; n_controls_ = 16 ONs, 64 ± 3 years, 5 females), group III (70–79 years old; n_NTG_ = 18 ONs, mean age: 74 ± 3 years, 7 females; n_controls_ = 18 ONs, 75 ± 3 years, 4 females) and group IV ( > 80 years old; n_NTG_ = 6 ONs, mean age: 83 ± 1 years, 3 males; n_controls_ = 6 ONs, 86 ± 2 years, 1 female) (Table 1).

**Table 1:** Summary of results for the NTG patients (*n* = 26).

Age category	Age (years)	Gender	IOP (mmHg)		VF (dB)		FR (CSF flow in SAS ON) (μm/ms)		FR (CSF flow in brain) (μm/ms)		FRR	
			OD	OS	OD	OS	OD	OS	OD	OS	OD	OS
**50–59** **y**.												
	51	M	13	13	1.25	1.25	356 ± 124	710 ± 115	384 ± 193	814 ± 517	0.50^a^	0.52
	52	M	17	17	9.5	7.5	207 ± 51	406 ± 123	274 ± 77	500 ± 161	0.53	0.61^a^
	52	M	13	12	2	11	367 ± 99	326 ± 65	628 ± 93	565 ± 96	0.58^a^	0.58
	53	M	13	12	2.0	11.0	367 ± 99	515 ± 96	326 ± 65	628 ± 93	^b^	0.52^a^
	54	M	13	13	1	1	195 ± 40	449 ± 85	148 ± 44	334 ± 116	0.46^a^	0.47
	54	M	14	15	2.0	19.0	303 ± 59	535 ± 67	364 ± 64	628 ± 154	^b^	0.59^a^
**60–69** **y**.												
	61	F	12	14	7.0	5.1	264 ± 50	527 ± 123	320 ± 100	537 ± 171	0.50^a^	0.59
	62	M	16	16	1	1.25	306 ± 87	852 ± 286	372 ± 44	595 ± 160	0.37	0.66^a^
	63	F	14	13	1	1	293 ± 71	479 ± 156	394 ± 164	901 ± 437	0.64^a^	0.48
	66	F	13	14	14.8	17.9	296 ± 126	519 ± 172	190 ± 32	503 ± 34	0.38	0.56^a^
	67	F	13	16	0.6	13.5	346 ± 76	533 ± 133	504 ± 83	814 ± 212	0.53^a^	0.55
	68	F	15	16	15.0	9.0	301 ± 49	560 ± 133	438 ± 144	522 ± 77	0.54	0.65^a^
	68	F	15	14	1.2	0.1	212 ± 40	332 ± 137	167 ± 12	376 ± 104	0.46^a^	0.69
	68	M	14	13	1.3	20.3	353 ± 89	560 ± 241	279 ± 35	582 ± 153	^b^	0.49^a^
**70–79** **y**.												
	71	F	12	16	1.7	2.3	175 ± 41	419 ± 170	228 ± 54	514 ± 140	0.46^a^	0.49
	71	F	5	12	11.2	15.2	273 ± 50	466 ± 117	168 ± 91	299 ± 79	0.60	0.58^a^
	72	F	9	12	3.2	6.3	352 ± 71	647 ± 142	416 ± 124	764 ± 131	0.55^a^	0.55
	73	M	17	14	9.5	19.3	189 ± 31	339 ± 67	184 ± 63	361 ± 127	0.56	0.51^a^
	75	F	18	12	3.2	15.1	320 ± 44	666 ± 291	291 ± 86	552 ± 106	0.50^a^	0.48
	75	F	14	16	17.0	11.5	343 ± 72	598 ± 149	256 ± 76	498 ± 93	0.59	0.51^a^
	76	F	10	11	25.2	18.37	315 ± 88	634 ± 115	277 ± 68	725 ± 171	0.51^a^	0.41
	77	M	10	10	0.9	10.9	396 ± 57	625 ± 94	300 ± 66	528 ± 118	0.59	0.64^a^
	78	F	10	10	1.0	0.8	432 ± 60	727 ± 92	277 ± 68	551 ± 123	0.59^a^	0.51
**> 80** **y**.												
	82	M	15	15	0.8	1.0	320 ± 101	646 ± 263	306 ± 46	587 ± 244	0.58^a^	0.59
	84	M	10	10	13.4	19.2	367 ± 86	566 ± 136	387 ± 90	647 ± 98	0.61	0.62^a^
	84	M	14	14	18.0	21.0	415 ± 94	657 ± 182	447 ± 180	844 ± 263	0.64^a^	0.52

*dB* decibel, *F* female, *FRR* flow-range ratio, *M* male, *NTG* normal tension glaucoma, *OD* oculus dexter (right eye), *OS* oculus sinister (left eye), *VF* visual field.

^a^for statistics every second OD and every second OS were chosen.

^b^Non-NTG eyes that have been excluded from the results (*n* = 3) are marked grey.

The flow velocity between the frontal lobe SAS and the SAS of the ON was calculated and presented in ratios. This was then compared between NTG’s and control’s age-categories.

### MRI

Images were acquired with a 3 T whole body magnet (Skyra; Siemens Healthcare, Erlangen, Germany) with a 32-channel head coil using Stejskal-Tanner diffusion sequence using following parameters: *b* = 50 s/mm^2^, TE/TR = 65/2000 ms, 6 slices, 1 mm slice thickness with acquisition time of 4.13 min, each slice acquired 120 times. Estimating the flow velocities of coherent moving particles through phase contrast images is described in detail in Boye et al. [11]. In-house code programmed in Matlab (MathWorks) was used for image analysis [11]. Shortly, the monopolar diffusion gradients of the diffusion sequence led to a constant phase shift for coherently moving particles. By using the *b*-values, the maximum encoded velocity (venc) before a phase wrap occurs can be solved and the phase shift can then be used to determine the flow velocity range. Since the phase shift of the diffusion sequence is highly irregular, results are presented as Flow Range Ratio (FRR), which allows the easy comparison between different groups.

### Statistics

Statistical analysis was performed with unpaired *t*-test performed by SPSS Statistics Software version 21 (IBM Corporation, Armonk, NY, USA) to compare the FRR results between the (i) NTG patients and healthy controls within the age groups, (ii) NTG patients within the age groups as well as (iii) healthy controls within the age groups. For both groups, every second OD and every second OS ON was chosen for statistics.

## Results

**Fig. 1:**
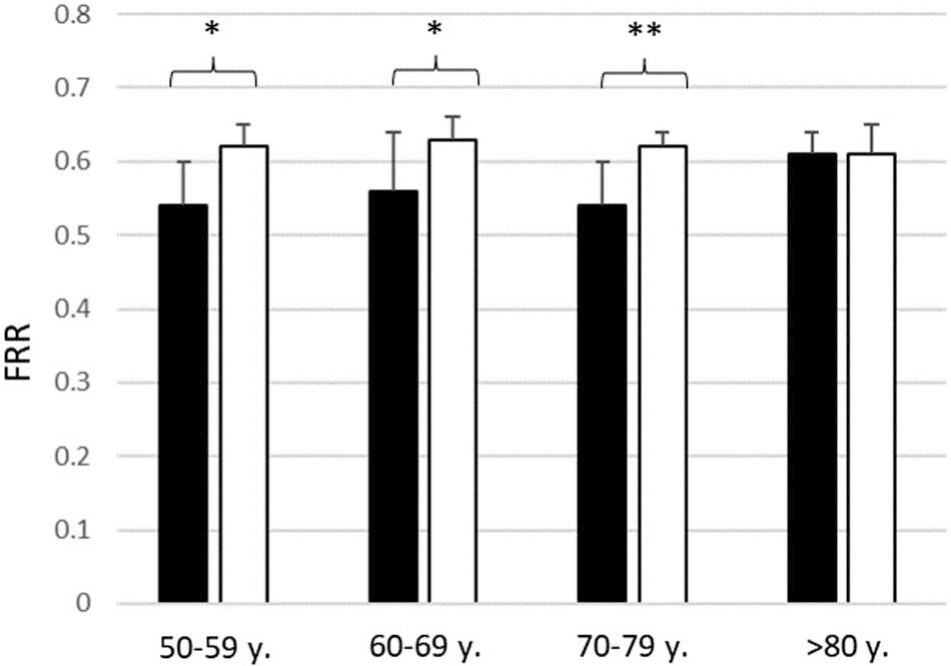
Statistically significant difference between the FRR of NTG patients when compared to the healthy volunteers. Subgroup comparison between patients with NTG (*n* = 26) and age-matched healthy volunteers (*n* = 26) based on their age: group I (50–59 years old, *n* = 6 eyes), group II (60–69 years old, *n* = 8 eyes), group III (70–79 years old, *n* = 9 eyes) and group IV ( > 80 years old, *n* = 3 eyes). Statistically significant difference marked with *(*p* < 0.05)/**(*p* < 0.001) between the FRR of NTG patients when compared to the healthy volunteers. Group IV is left out of the statistical comparisons due to a small subject size.

The mean FRR for age groups, when considering 1 eye per person, were (I) 0.54 ± 0.06 (95% CI [0.42, 0.66]) and 0.62 ± 0.03 (95% CI [0.56, 0.68]), *n* = 6, *p* < 0.05, (II) 0.56 ± 0.08 (95% CI [0.40, 0.72]) and 0.63 ± 0.03 (95% CI [0.57, 0.69], *n* = 8, *p* < 0.05), (III) 0.54 ± 0.06 (95% CI [0.42, 0.66]) and 0.62 ± 0.02 (95% CI [0.58, 0.66], *n* = 9, *p* < 0.001) and (IV) 0.61 ± 0.03 (95% CI [0.55, 0.68]) and 0.61 ± 0.04 (95% CI [0.53, 0.69]), for patients with NTG and healthy controls, respectively (statistically significant difference given in brackets: Fig. 1, Tables 1 and 2).

**Table 2:** Flow-range ratio (FRR) analysis results for healthy volunteers (*n* = 26).

Age category	Age (years)	Gender	FR (CSF flow in SAS ON) (μm/ms)		FR (CSF flow in brain) (μm/ms)		FR (CSF flow in SAS ON) (μm/ms)	
			OD	OS	OD	OS	OD	OS
**50–59 y**.								
	50	F	323 ± 68	330 ± 42	488 ± 122	529 ± 37	0.62^a^	0.62
	51	M	403 ± 40	520 ± 106	524 ± 41	640 ± 68	0.74	0.66^a^
	55	M	235 ± 54	375 ± 118	328 ± 48	525 ± 96	0.58^a^	0.61
	57	F	278 ± 69	464 ± 112	302 ± 96	477 ± 171	0.60	0.64^a^
	53	F	285 ± 123	482 ± 205	295 ± 58	484 ± 84	0.60^a^	0.61
	59	M	240 ± 65	399 ± 100	364 ± 68	582 ± 109	0.60	0.63^a^
**60–69 y**.								
	60	F	292 ± 73	325 ± 70	455 ± 124	514 ± 178	0.63^a^	0.63
	60	F	425 ± 67	704 ± 74	476 ± 136	798 ± 226	0.60	0.61^a^
	61	F	300 ± 91	391 ± 138	465 ± 111	653 ± 108	0.61^a^	0.52
	62	M	605 ± 55	987 ± 109	238 ± 47	378 ± 53	0.61	0.63^a^
	64	M	352 ± 96	471 ± 152	470 ± 114	593 ± 115	0.64^a^	0.67
	66	F	331 ± 68	624 ± 109	401 ± 67	531 ± 103	0.56	0.59^a^
	66	F	397 ± 173	652 ± 273	385 ± 108	623 ± 189	0.61^a^	0.62
	69	M	352 ± 68	594 ± 42	450 ± 96	557 ± 71	0.66	0.68^a^
**70–79 y**.								
	70	M	364 ± 92	585 ± 185	398 ± 53	618 ± 92	0.63^a^	0.64
	73	M	576 ± 131	938 ± 205	769 ± 121	1200 ± 216	0.61	0.64^a^
	73	F	524 ± 150	851 ± 240	421 ± 130	673 ± 218	0.61^a^	0.63
	73	F	241 ± 54	393 ± 82	383 ± 176	646 ± 315	0.61	0.60^a^
	74	M	686 ± 83	446 ± 176	566 ± 114	868 ± 187	0.60^a^	0.66
	75	M	497 ± 100	814 ± 162	291 ± 95	485 ± 116	0.61	0.60^a^
	78	M	345 ± 82	457 ± 131	484 ± 156	745 ± 253	0.64^a^	0.66
	79	F	385 ± 91	292 ± 152	641 ± 79	840 ± 194	0.63	0.60^a^
	79	F	353 ± 139	544 ± 200	348 ± 129	624 ± 275	0.65^a^	0.59
**> 80 y**.								
	83	M	424 ± 145	684 ± 220	518 ± 172	872 ± 290	0.62^a^	0.60
	87	M	237 ± 74	375 ± 118	326 ± 48	525 ± 96	0.59	0.64^a^
	87	F	300 ± 36	548 ± 107	426 ± 140	643 ± 113	0.56^a^	0.65

*CSF* cerebrospinal fluid, *F* female, *FR ON SAS* flow range (min–max) in optic nerve subarachnoid space, *M* male, *OD* oculus dexter

(right eye), *OS* oculus sinister (left eye).

^a^for statistics every second OD and every second OS were chosen.

For pooled data (including all the age groups), the difference between the FRR in NTG patients (0.55 ± 0.06, 95% CI [0.43, 0.67]) and healthy controls (0.62 ± 0.03, 95% CI [0.56, 0.68]) was statistically significant (*n* = 26, *p* < 0.0001).

When comparing the FRR by age categories no statistically significant difference was found between the age categories neither for NTG patients nor for the healthy controls. Groups IV were left out of the statistical comparisons due to a small subject size.

## Discussion

This study performed with diffusion weighted MRI demonstrates a significantly reduced CSF flow range ratio (FRR) in patients with NTG compared to age-matched controls without a history of glaucoma. While the CSF flow range in NTGs was the same in the age groups I, II and III and greater in group IV, in age-matched healthy controls a slight decrease from group I to IV was observed.

Calculation of the flow range ratio (FRR) on diffusion-weighted MRI scans between the SAS of the frontal lobe and the SAS of the SAS of the ON reflects the flow velocity of the CSF. These measurements therefore allow the assessment of CSF flow dynamics in the SAS of the ON in a non-invasive way.

The pathophysiology of the optic neuropathy in NTG is poorly understood. Although reduction of intraocular pressure can slow the progression of the disease, a substantial number of patients progress despite successfully lowered IOP [5]. Alternative pathophysiological explanations are therefore under discussion [4, 7]. Several recent studies indicated disturbed CSF dynamics in the SAS of the ON with pathological changes in pressure and CSF composition [7, 10]. In a large series of NTG patients using computer tomography assisted cisternography a gradual reduction of contrast loaded CSF from intracranially to the bulbar portion of the ON behind the lamina cribrosa was demonstrated in contrast to the control group [10]. In accordance with these findings the current study demonstrates a reduced CSF flow velocity in the SAS of the ON in the NTG group compared to a non NTG cohort.

At least two possible mechanisms can explain reduced CSF flow within the SAS along the ON in NTG patients: (i) compartmentalisation of the optic nerve sheath and (ii) low CSF pressure. The ON SAS is bridged on its whole length with trabeculae and septae [15]. These anatomical structures as well as the pia and the arachnoid layer are covered with meningothelial cells which display mechanosensitive characteristics. They proliferate and growth if the external pressure is being elevated or if oxidative stress is applied [16]. Such growth and proliferation lead to a remodelling of the SAS and a reduction of the free space that allows CSF to flow. The CSF pressure within an optic nerve sheath compartment is unknown but, due to its compartmentalisation, likely independent from that intracranially. Low intracranial pressure, as suggested by some groups who have measured lower lumbar CSF pressure in patients with NTG, may also play a role [17]. Decreased CSF pressure directed from the basal cistern to the ON SAS, particularly in combination with a narrower OC, could therefore be another explanation for lower CSF flow velocity within the ON SAS in NTG patients. However, whether the intracranial CSF pressure is reduced in patients with NTG remains controversial [18].

In the current study, the CSF flow range in NTG patients was the same in the age groups from 50 to 79 years (groups I, II and III) while it was larger in the age group over 80 years (group IV). If optic nerve sheath compartmentalisation with impaired CSF turnover plays a role in the pathophysiology of NTG, the severity of compartmentalisation may be the most important factor. The exact mechanism leading to ON sheath compartment is however not yet revealed. As it is increasingly found in patients with elevated CSF pressure and in patients with a history of meningitis, age-independent pressure gradients and inflammatory processes seem to play a role [19]. The larger CSF flow range in NTG patients over 80 may be explained by the small number (*n* = 3) of patients in this group.

There is a growing body of evidence that CSF dynamics seems to become impaired with age which plays a crucial role in brain health in older people [20]. However, in the current study we did not find a clear decrease of the CSF flow range with age, which may be due to the relatively small numbers in each age group. There are only few studies that have systematically examined CSF pressure and age. While some studies [21] did not find a relationship between age and CSF pressure, a retrospective study demonstrated in 12,118 patients a sustained and significant reduction of CSF pressure beginning in the 6th decade [22]. A lower CSF pressure will consequently lead to a lower CSF flow velocity. CSF pressure is usually measured by lumbar puncture. As lumbar puncture is performed quite remote from the ON SAS it is not clear whether the lumbar CSF pressure equals the CSF pressure in the ON SAS and whether the CSF pressure is lower at all in the older controls.

Adequate CSF flow is essential for the delivery of neurotropic factors, electrolytes, neurotransmitters and hormones to neurons, axons, and glial cells in the central nervous system (CNS). However, its role as a clearing system of the CNS is just as important. Several neurodegenerative diseases, such as Alzheimers disease, Parkinsons disease, Lew-body -dementia and frontotemporal dementia are linked to a reduced clearage of abetalipoprotein, alfasynuclean or Tauproteins and other biomarkers [23]. Stagnant CSF causes an accumulation of such biomarkers of neurodegeneration. CSF samples from a compartmented ON SAS of NTG patients have demonstrated elevated levels of L-PGDS (a multifunctional prostaglandine synthetase), indicating CSF stagnation in the ON SAS [24]. However, future studies need to investigate how and to what extent CSF stagnation in the optic nerve SAS leads to the optic nerve damage as seen in patients with NTG.

There are some limitations of this study: First, as the CSF flow within the ON is dependent on the RR interval in ECG-curve of the heart, the RR interval was used to report our results as ‘flow range’, and not as flow, since the phase offset. Ratio is not dependent on the offset, therefore, making it possible to compare values between the measurements. Second, the number of subjects was relatively small in the age-categorised subgroups, particularly in group IV, which was therefore left out of the statistical comparisons.

In summary, CSF flow range was significantly reduced in patients with NTG compared to age-matched non glaucomatous controls. CSF flow studies applying non-invasive flow rate measuring could be used as an addition to intraocular pressure measurement in therapy refractive NTG patients.

## Summary

### What was known before

- Age is one of the main risk factors for NTG.
- CSF flow and thus CSF turnover along the optic nerve can become reduced in patients with NTG.
- Patients and controls were divided in age groups in order to investigate a possible influence of age on CSF flow rates.

### What this study adds

- CSF Flow was significantly reduced in NTGs in different age categories compared to age-matched controls without any significant differences within the age groups themselves.
- Reduced CSF flow dynamics might be part of the underlying neurodegenerative process of NTG.

## Supplementary information

Reporting Checklist

## Data Availability

Data is freely available for non-commercial purposes and can be requested from the corresponding author.
